# Value of Multitracer Imaging in Hepatocellular Carcinomas with Different Metastatic Potential

**DOI:** 10.1007/s11307-026-02088-7

**Published:** 2026-02-24

**Authors:** Tingting He, Cong Zhang, Jinming Zhang, Xiaojun Zhang, Ruimin Wang

**Affiliations:** 1 Beijing Universal Dicom Medical Imaging Diagnostic Center, Beijing, 100039 China; 2 Department of Nuclear Medicine, The First Medical Center, Chinese PLA General Hospital, Haidian District, No. 28 Fuxing Road, Beijing, 100853 China

**Keywords:** [^18^F]FDG, [^18^F]FLT, [^18^F]ACE, Hepatocellular carcinoma, Metastasis, PET imaging, Biological heterogeneity, Prognostic marker

## Abstract

**Background:**

Hepatocellular carcinoma (HCC) shows marked heterogeneity and varying metastatic potential, challenging prognosis and treatment. This study evaluated multitracer PET imaging with [^18^F]FDG, [^18^F]FLT, and [^18^F]ACE to differentiate HCCs by metastatic behavior and explored its prognostic relevance.

**Methods:**

Four human HCC cell lines with varying metastatic potential (HepG2, QGY7701, MHCC97-H, MHCC97-L) were assessed for in vitro tracer uptake, proliferation, and invasion. Subcutaneous and spontaneous metastasis models were established in nude mice. Tumor uptake of tracers was quantified via microPET/CT. mRNA expression of MMP9 and VEGFR-2 and survival were analyzed. Fisher’s classifier was applied to compare single-, dual-, and triple-tracer datasets with cross-validation.

**Results:**

Tracer uptake significantly differed among cell lines (*p* < 0.001). The triple-tracer model ([^18^F]FDG + [ ^18^F]FLT + [^18^F]ACE) yielded the lowest classification error rate (0.074) compared with dual tracers (0.085–0.362). In vivo, [^18^F]FDG uptake correlated with metastatic potential, MMP9 and VEGFR-2 expression (r = 0.770–0.830, *p* < 0.05), and inversely with survival (r = − 0.726, *p* = 0.005). There is a significant difference in FDG uptake values between high-metastatic and low-metastatic cell lines as well as in tumor model.[^18^F]FLT uptake showed moderate correlation with biomarkers but not with survival, while [^18^F]ACE had no significant discriminative value.

**Conclusions:**

A multiparameter (multitracer) classification model had superior ability to discriminate four HCC cell lines with different biological behavior compared with dual tracers or single tracer through in vitro cell uptake experiment. [1⁸F]FDG uptake can distinguish between tumor models with high and low metastatic potential, and [1⁸F]FDG uptake can predict the survival time in metastatic models. [^18^F]FDG uptake probably be noninvasive prognostic marker, reflecting tumor heterogeneity and correlating with survival and metastasis-related biomarkers. This approach may aid in clinical stratification and personalized management of HCC.

## Introduction

Hepatocellular carcinoma (HCC) is a malignant tumor that is common worldwide and has a high prevalence in Asia. In China, the incidence rate of liver cancer is 8.6%, and its 5-year survival rate is 14.4%. Although progress in therapeutic procedures, including surgical intervention, has increased the 5-year survival rate for HCC, the high incidence of metastasis or recurrence (50%−70%) after resection still results in poor prognosis[1]. Therefore, metastasis remains one of the major obstacles in the treatment of HCC.

Tumor metastasis is a complex biological process involving multiple steps such as cell adhesion, extracellular matrix changes, tumor angiogenesis, and genetic/epigenetic alterations as a hallmark of tumor heterogeneity. Previous studies have reported that several metastasis-related biomarkers that play an important role in these steps, such as matrix metalloproteinases (MMPs) and vascular endothelial growth factor receptor (VEGFR) [2, 3] can serve as targets for antimetastasis intervention and as biomarkers [4–6] to help predict prognosis. However, these experiments were performed in vitro, and might not be easily translated into the clinical setting. In vivo monitoring of metastasis and other biological behaviors is important because variations in the biological behaviors of tumors reflect recurrence and guide the development of individualized therapy. It has been suggested that, since metastasis is a complicated bioprocess, imaging with multiple tracers that each provide insight into certain biological behaviours of a tumor inside a living organism might be more informative for characterizing the tumor.

Positron emission tomography (PET) is widely used in the detection of HCC and its metastasis [7, 8] and has been extended to preclinical research. PET allows non-invasive in *vivo* imaging and quantification of biological processes such as metabolism, cell proliferation, apoptosis, and lipid synthesis, and can therefore be used to monitor the biological behaviors of tumors [9, 10]. [^18^F]Fluorodeoxyglucose ([^18^F]FDG) is the most commonly used tracer in clinical PET imaging, and its uptake has been reported to correlate with tumor differentiation level and may even serve as an independent prognostic factor in HCC and predict tumor recurrence [11]. [^18^F]FDG also has relatively high sensitivity for the detection of extrahepatic metastasis of HCC [12].

In addition, the thymidine analogue [^18^F]fluorothymidine ([^18^F]FLT) has been introduced for imaging tumor proliferation [9, 10]. [^18^F]FLT is trapped within the cytosol after being monophosphorylated by thymidine kinase-1, a principal enzyme in the salvage pathway of DNA synthesis. A few reports found that the [^18^F]FLT could monitor the tumor response after therapy and might be an important prognostic factor for clinical HCC patients [10, 13]. [^18^F]Fluoroacetate ([^18^F]ACE) is also being evaluated as a PET agent for tumor imaging. Fluoroacetate, an analog of acetate, is metabolized to fluoroacetyl-CoA and then to fluorocitrate, which cannot be further metabolized to CO_2_ and water [14]. As a result, fluorocitrate is trapped in the cell in proportion to the level of oxidative metabolism and is expected to be a promising oncologic PET tracer [15].

As these three tracers provide different information on tumor cells, we hypothesized that a multitracer ([^18^F]FDG, [^18^F]FLT, and [^18^F]ACE) model might better reflect tumor behaviours in vivo. The present study therefore examines whether multitracer imaging could more precisely reflect the behaviours of different HCCs in vivo and thereby better differentiate and predict the metastatic potential of HCCs in vitro and in vivo, and how to better demonstrate and make visually interpretable the multiple fold of information gained from the experiment.

## Methods

### Target Cell lines

The human HCC cell lines MHCC97-H and MHCC97-L purchased from Institute of Liver Cancer, Fudan University, HepG2, and QGY7701were provided by the Hepatobiliary Surgery Laboratory, Chinese PLA General Hospital. MHCC97-H and MHCC97-L are clonal cells derived from the parental cell line MHCC97 and have a similar genetic background but different metastatic potential [16]. Spontaneous metastasis assays have revealed that MHCC97-H has a higher metastatic potential than MHCC97-L [17]. HepG2 and QGY7701 cells had different tumorigenicity and invasion [18, 19].

### Radiotracers

[^18^F]FDG was automatically synthesized with a conventional synthesizer module used in clinical work, as a glucose analog measuring glucose uptake. [^18^F]FLT was synthesized according to the procedure described by Oh et al. [20] with modifications, and [^18^F]ACE was synthesized on a FDG synthesizer. All procedures had been approved by the Radiopharmaceutical Administration and complied with Good Manufacturing Practices guidelines. The three tracers were pyrogen-free, qualified for clinical use, and radiochemical purity are > 95%.

### Cell Culture and Cell Grow Curve

MHCC97-H and MHCC97-L cells were cultured in high-glucose Dulbecco’s modified Eagle’s medium (DMEM; PAA Company, Austria) supplemented with 10% fetal bovine serum (PAA Company, Austria)) and l-glutamine at 37 °C in a humidified atmosphere of 5% CO_2_. HepG2 and QGY7701 were cultured in RPMI1640 at 37 °C in a humidified atmosphere of 5% CO_2_. Cells were passaged every 3–4 days.

### Matrigel Invasion Assay

To assess cell invasion, cells were seeded into Matrigel invasion chambers. Firstly, the membranes of Costa Transwell (Corning company) filter inserts were coated with Matrigel (BD Biosciences: Sparks, MD, USA) diluted with DMEM at the ratio of 1:1, cells were suspended in DMEM and added to the upper chamber of the transwell plates (2 × 10^5^ cells/well). The plates were incubated at 37 °C in a humidified atmosphere of 5% CO2. After incubation for 48 h, chamber was taken out and the cells that failed to penetrate the filters were gently removed by cotton swabs. The invading cells in the membrane were stained with 0.1% crystal violet and cleaned with PBS for 3 times. The average number of invasive cells was counted in three random high-power fields (× 400). After that, Decolorizing with 33% acetic acid for 20 min, the crystal violet was completely eluted. The OD value of the eluent was measured at 562 nm on the enzyme label, which indirectly reflected the number of invasive cells.

### Subcutaneous Tumor-bearing Model

Male BALB/C *nu*/*nu* mice (6–8 weeks old) were obtained from the Animal Experiment Center of Chinese PLA General Hospital and maintained under specific pathogen-free conditions with a standardized light/dark cycle and ad libitum access to food and water. In this study, male mice were selected as the experimental subjects, primarily based on the epidemiological evidence that males exhibit a significantly higher incidence rate of liver cancer. Subcutaneous tumors were established by injection of approximately 5 × 10^6^ cells (in 100 μL PBS) into the left posterior leg for MHCC97-H and the right posterior leg for MHCC97-L (*n* = 10 per group). Tumor growth was determined by caliper measurements using the formula *V* = 1/2(*L* × *S*^2^), where *V* is the volume, *L* is the long diameter, and *S* is the short diameter. MicroPET scans were performed until the tumor reached a diameter of 8–10 mm.

### Spontaneous Metastasis Model

Male BALB/C nu/nu mice (6–8 weeks old) were obtained from the Animal Experiment Center of Chinese PLA General Hospital. Thirty mice were anesthetized with 0.6% pentobarbital sodium (45–55 mg/kg), and underwent laparotomy via a midline abdominal incision or a left upper flank incision. To reveal the left lobe of the liver, and extrude the left lobe of the liver with instruments outside the incision. 5 × 10^6^ MHCC97-H or MHCC97-L cells/50 μl in FBS was injected into the left upper lobe slowly (15 mice for each cell line). After injection, adding biogel to prevent liquid exudation and wash with 75% alcohol. After observing inactive bleeding, the liver was sent to the abdomen and the abdomen was gradually closed. Mice were returned to their cages. The animal model was imaged 6 weeks after surgery and monitored for survival and metastatic lesions. Euthanasia must be performed immediately if the mice meet any of the following conditions: tumor accounts for 10%–15% of body weight, diameter > 20 mm, or presents with ulceration/infection; > 15% sustained body weight loss accompanied by lethargy and reduced food intake. After confirming the death of the mice, the carcasses were disposed of in strict accordance with the laboratory biosafety regulations and environmental protection requirements.

### Cell Uptake Assay

Cells were plated in 24-well plates (2–4 × 10^5^ cells/well) and cultured for 2–3 days. The day before the experiment, culture the cells in serum-free medium to induce cell starvation.100 μCi [^18^F]FDG, [^18^F]ACE or [^18^F]FLT were diluted with PBS respectively, Maintain a stable count between 40,000 and 100,000 counts per second (cps), with 50,000 cps being optima in test tube. Then added to wells and incubated for 60 min at 37 °C. After incubation, cells were washed by PBS twice to remove free tracer. Cells were detached from the well with 0.25% trypsin and accumulated in same volume of PBS. Cellular tracer uptake was assessed in a single-well gamma counter. The harvested cell number was normalized to 10^5^ cells. All data were collected in triplicate. Calculate %ID/10^5^ cells: (Net intracellular cps/Total cps) × 100% × (10^5^/Cell number).

### MicroPET Imaging

PET and imaging analyses were performed using Explore VISTA MicroPET (GE, USA). Tracers were injected into the tail vein of the experimental animals at a dose of 18.5 MBq (500 μCi). At 50 min after tracer injection the mice were anesthetized with 1% chloral hydrate and placed on the examination bed for 10 min for static imaging acquisition 1 h after injection of tracer. For quantification of tumor uptake, ImageJ software was employed on the reconstructed images. Regions of interest (ROIs) were drawn on the tumor and on skeletal muscle in the thigh muscle as background on three consecutive coronal slices encompassing the maximum tumor uptake [21]. For each mouse, the mean counts within the tumor ROI and the mean counts within the muscle ROI were measured on each slice and averaged. The tumor-to-normal uptake ratio (T/NT) was then calculated as the ratio of tumor mean counts to muscle mean counts (T/NT = tumor/muscle). For the subcutaneous tumor-bearing model, PET imaging was performed on separate nude mice for each tracer; whereas for the spontaneous metastasis model, the imaging was conducted on the same nude mice with each tracer (Table 1).

**Table 1:** Summary of experimental design and PET imaging parameters for subcutaneous and metastatic HCC models

Model Type	Cell Line	Radiotracer	No. of Mice (n)	Imaging Checkpoint
Subcutaneous	MHCC97 H	[^18^F] FDG	6	Tumor diameter: 0.8–1.0 cm
		[^18^F] FLT	8	
		[^18^F] ACE	10	
	MHCC97 L	[^18^F] FDG	10	Tumor diameter: 0.8–1.0 cm
		[^18^F] FLT	7	
		[^18^F] ACE	9	
Metastatic	MHCC97 H	[^18^F] FDG	8	6 weeks post establishment
		[^18^F] FLT	4^**a**^	
	MHCC97 L	[^18^F] FDG	5^**b**^	6 weeks post establishment
		[^18^F] FLT	7	

### Real-Time PCR (RT-PCR)

RT-PCR analysis was performed using tissue samples of MHCC97-H and MHCC97-L tumors as described by Révillion et al. [22]. Total RNA was extracted and reverse transcribed. The resulting cDNA was subjected to RT-PCR using primers for MMP9 (5′-CGCAGACATCGTCATCCAGTT-3′ and 5′-ATGGGCGTCTCCCTGAATG-3′) and VEGFR-2 (5'-GTGTTCTTCGAGTTGGGCTAAAG-3' and 5'-ATAAAGGATCAGCCTGGGAGACA-3'). β-actin was used as an internal control. Data were analyzed according to the comparative Ct method and normalized to β-actin expression in each sample (relative quantity, 2^^−ΔΔCt^).

### Statistical Analysis

An independent-samples *t* test and one-way ANOVA (Tamhane's T2 test) were used to compare differences in tumor growth and uptake of [^18^F]FDG, [^18^F]ACE, or [^18^F]FLT. Kaplan–Meier survival curves were plotted to compare the survival time of metastatic models. The log-rank test was used to assess the difference in survival time between the two groups. Fisher’s exact test was used to compare differences in metastatic potential. The correlation of tumor uptake with biomarkers or survival was analyzed using Pearson’s correlation. SPSS20.0 was used for statistical analysis and *p* < 0.05 was considered statistically significant. The results were expressed as mean ± standard deviation. A classification model was established for cell data analysis using Matlab (version: R2010b). First, three-feature data (uptake rate of [^18^F]FDG, [^18^F]ACE, and [^18^F]FLT) was subjected to Multidimensional Scaling, which is a dimension reduction method, because three features cannot display in 2D figures, and two-feature data and three-feature data were both standardized to [0,1]. Fisher’s classifier was used to classify four HCC cell lines using a 3-feature dataset ([^18^F]FDG + [^18^F]FLT + [^18^F]ACE) or 2-feature dataset ([^18^F]FDG + [^18^F]FLT, [^18^F]FDG + [^18^F]ACE, [^18^F]FLT + [^18^F]ACE). The dataset was randomly divided a set for training and a set for testing. Two-fold cross validation was performed on the 3-feature dataset and 2-feature datasets 100 times and the mean error rate was shown as the analysis result. The training set accounts for 50% and the validation set accounts for 50%. A lower error rate indicates higher reliability of predictions.

## Results

### Cell Growth and Invasion Ability

**Fig. 1:**
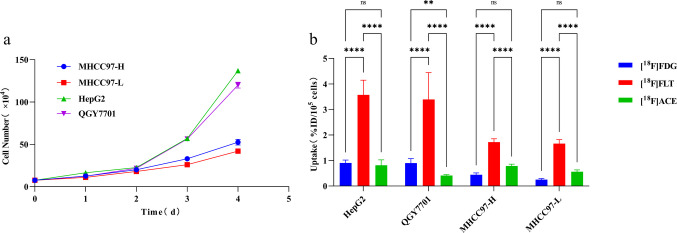
Growth kinetics and multitracer uptake profiles of HCC cell lines with varied metastatic potential. **a** Cell proliferation curves, **b** [^18^F]FDG, [^18^F]FLT and [^18^F]ACE uptake in four hepatocellular carcinoma cell lines (HepG2, QGY7701, MHCC97-H, MHCC97-L). Data reflect mean ± SD of triplicate experiments

**Fig. 2:**
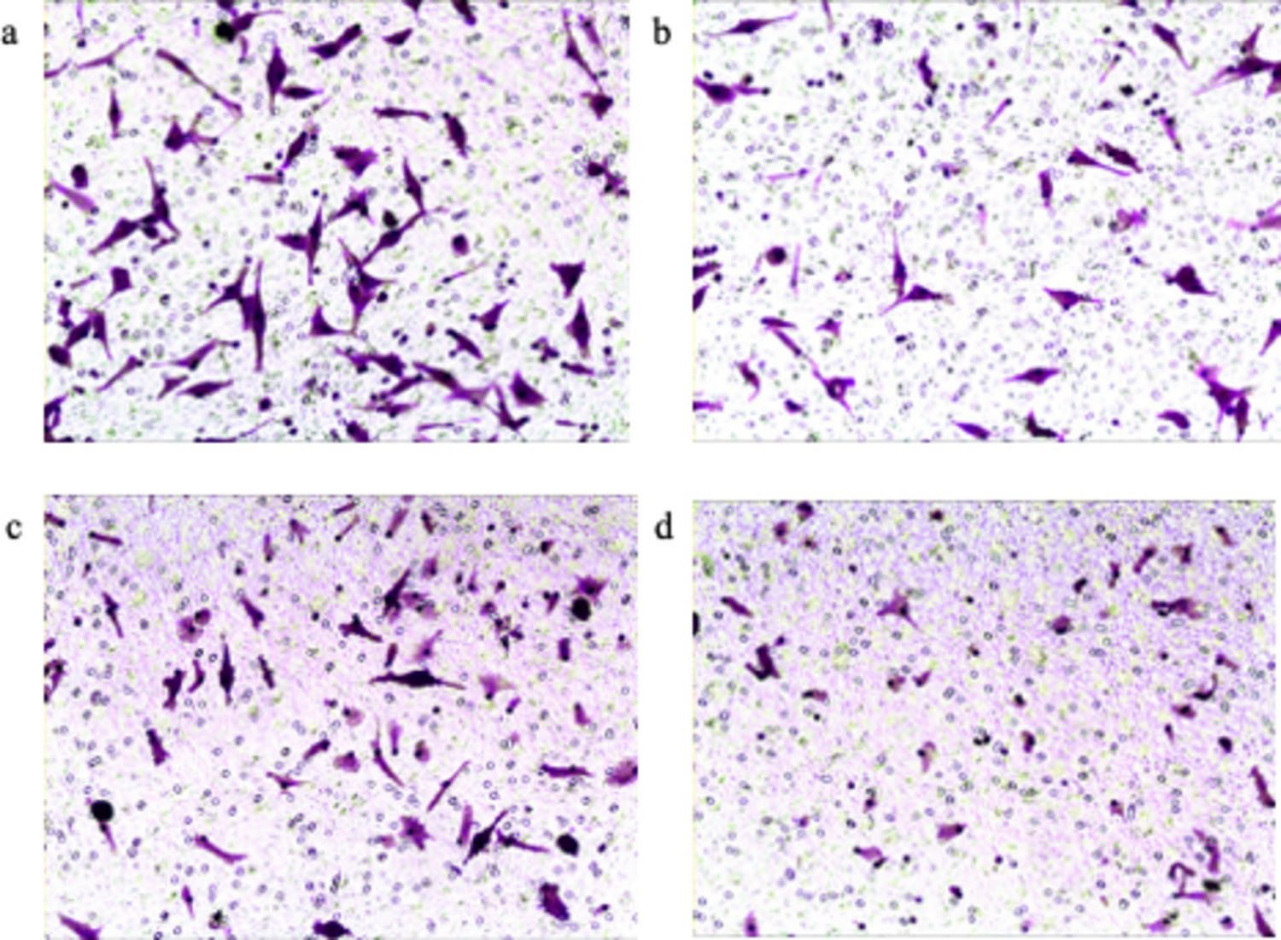
Crystal violet staining of four tumor cells (× 400). **a** HepG2. **b** QGY7701. **c** MHCC97-H. **d** MHCC97-L

HepG2 and QGY7701 cells grew faster than MHCC97-H and MHCC97-L cells **(**Fig. 1a). The four cell lines also differed in invasion activity (F = 36.85, *p* < 0.001): the OD values of HepG2 (0.433 ± 0.025, *p* < 0.001), QGY7701 (0.347 ± 0.032, *p* = 0.001), and MHCC97-H (0.320 ± 0.036, *p* = 0.003) were significantly higher than those of MHCC97-L (0.187 ± 0.021)(Fig. 2).

### [^18^F]FDG, [^18^F]FLT, and [^18^F]ACE Uptake By Cell Lines

**Fig. 3:**
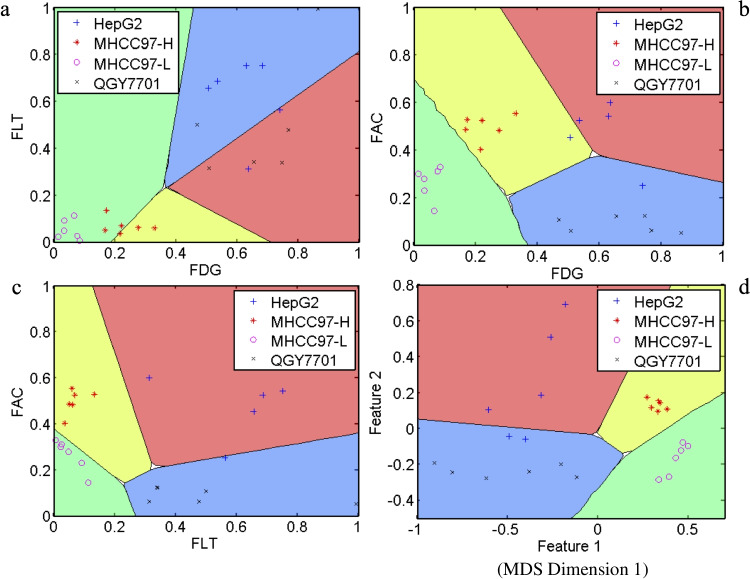
The testing dataset using Fisher’s classifier. **a** Testing dataset on 2 features ([^18^F]FDG + [^18^F]FLT), error rate 0.362. **b** Testing dataset on 2 features ([^18^F]FDG + [^18^F]ACE), error rate 0.085. **c** Testing dataset on 2 features ([^18^F]ACE + [^18^F]FLT), error rate 0.116. **d** Testing dataset on 3 features ([^18^F]FDG + [^18^F]ACE + [^18^F]FLT), error rate 0.074. Feature 1 (X-axis) represents the dimension with the greatest variation in distances between data points, and Feature 2 (Y-axis) represents the dimension with the second-greatest variation

The cell lines showed significant variation in [^18^F]FDG, [^18^F]FLT, and [^18^F]ACE uptake (Table 2). [^18^F]FDG uptake of HepG2 (0.902 ± 0.117) and QGY7701 (0.897 ± 0.174) cells was higher than that of MHCC97-H (0.442 ± 0.072, *p* < 0.001) and MHCC97-L (0.252 ± 0.043, *p* < 0.001) (Fig. 1b). [^18^F]FLT uptake of HepG2 (3.569 ± 0.579) and QGY7701 (3.393 ± 1.049) cells was also higher than that of MHCC97-H (1.720 ± 0.132, *p* = 0.001) and MHCC97-L (1.662 ± 0.157, *p* = 0.001) (Fig. 1b). However, the [^18^F]ACE uptake pattern was different from that of [^18^F]FDG and [^18^F]FLT; uptake of [^18^F]ACE was high in HepG2 (0.801 ± 0.218) and MHCC97-H (0.787 ± 0.071) and low in QGY7701 (0.413 ± 0.032, *p* < 0.001) and MHCC97-L (0.559 ± 0.072, *p* < 0.001) (Fig. 1b). Higher [^18^F]FDG or [^18^F]FLT uptake correlated well with more rapid cell growth (Fig. 1). For the classification model, the dataset was randomly divided into two groups, one for training and the other for testing. Fisher’s classifier (Fig. 3) indicated that the error rate was 0.362 ([^18^F]FDG + [^18^F]FLT), 0.085 ([^18^F]FDG + [^18^F]ACE), and 0.116 ([^18^F]ACE + [^18^F]FLT) for 2-feature datasets, compared with 0.074 for the 3-feature dataset ([^18^F]FDG + [^18^F]FLT + [^18^F]ACE).

**Table 2:** Pairwise comparisons of tracer uptake among four hepatocellular carcinoma cell lines

		Tracer	
Comparison Group	FDG	FLT	ACE
HepG2 vs QGY7701	(*p* = 1.000)	(*p* = 0.997)	*(*p* = 0.001)
HepG2 vs MHCC97-H	*(*p* = 0.001)	*(*p* = 0.001)	(*p* = 0.998)
HepG2 vs MHCC97-L	*(*p* = 0.001)	*(*p* = 0.001)	*(*p* = 0.008)
QGY7701 vs MHCC97-H	*(*p* = 0.001)	*(*p* = 0.001)	*(*p* = 0.001)
QGY7701 vs MHCC97-L	*(*p* = 0.001)	*(*p* = 0.001)	*(*p* = 0.001)
MHCC97-H vs MHCC97-L	*(*p* = 0.001)	(*p* = 0.917)	*(*p* = 0.001)

### Metastatic Potential and Survival

**Fig. 4:**
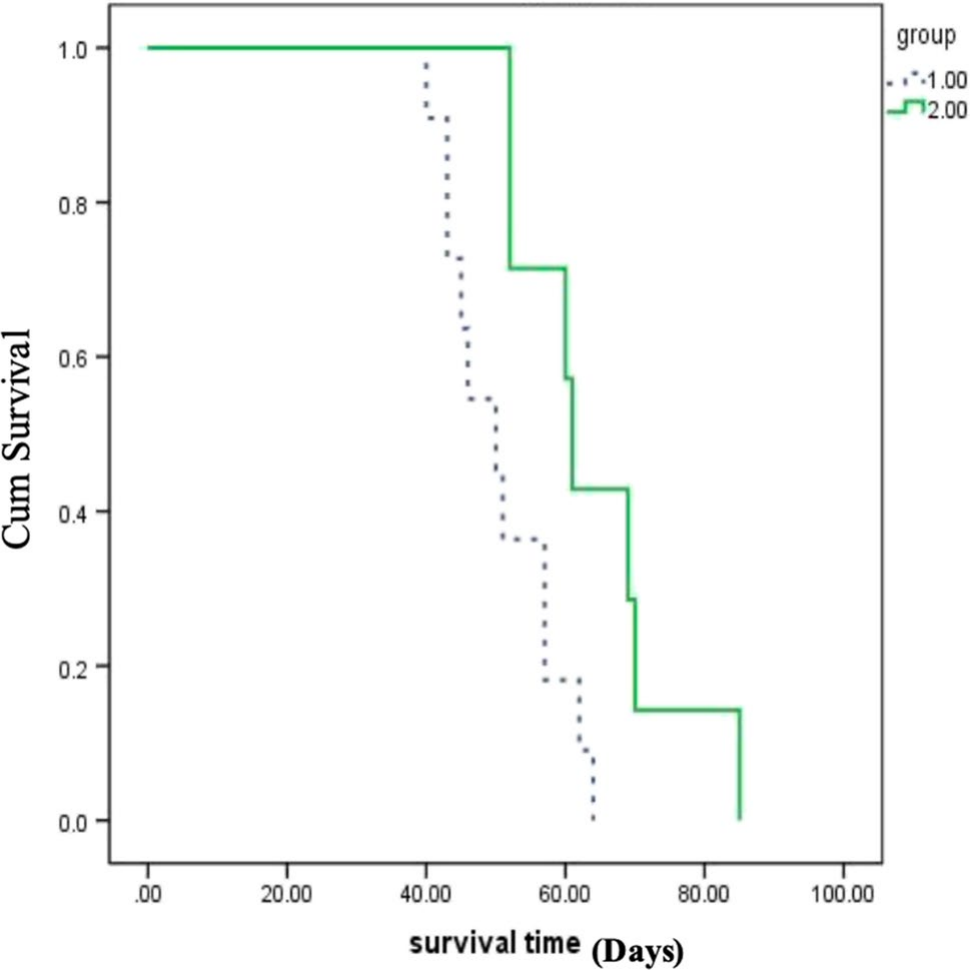
Survival curve analysis of MHCC97-H and MHCC97-L metastatic models. Group 1, MHCC97-H mice; group 2, MHCC97-L mice. The survival function of group 1 was higher than that of group 2, Χ2 = 5.761, *p* = 0.016

The metastatic potential of MHCC97-H and MHCC97-L cells was described by metastatic incidence. Metastatic lesions developed in 11 of 14 mice injected with MHCC97-H compared with 7 of 14 mice injected with MHCC97-L. The metastatic range was predominantly the liver, abdominal cavity, and diaphragm. Metastatic incidence was significantly different between MHCC97-H and MHCC9-L (Fisher’s exact test, *p* = 0.046). In the metastatic model via liver injection, the survival time of MHCC97-H mice (50.727 ± 2.472 d, n = 11) was shorter than that of MHCC97-L mice (64.143 ± 4.405 d, n = 7) without any intervention (χ2 = 5.761, df = 1, *p* = 0.016; Fig. 4).

### Tumor Growth

There was a significant difference in the growth of four xenografts in the mouse model. The growth rates of HepG2 and QGY7701 xenografts were faster than those of MHCC97-H and MHCC97-L xenograft, and the volumes of four tumors (HepG2: 0.709 ± 0.181cm^3^, QGY7701:0.532 ± 0.146 cm^3^, MHCC97-H:0.392 ± 0.069 cm^3^, MHCC97-L:0.229 ± 0.102 cm^3^) were significantly different at 2 weeks (F = 9.614, *p* < 0.001).

### MicroPET Imaging Analysis of Tumor-bearing Model

**Fig. 5:**
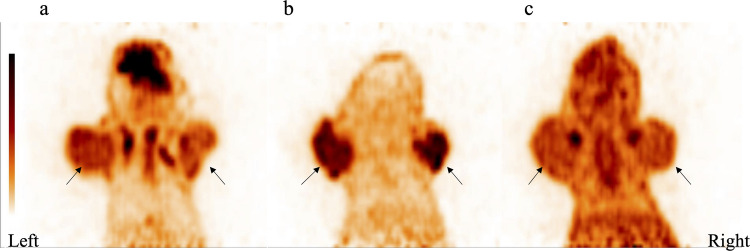
[^18^F]FDG, [^18^F]FLT, and [^18^F]ACE microPET images of MHCC97-H and MHCC97-L tumors. **a** The uptake of [^18^F]FDG in MHCC97-H tumors (left) was higher than that in MHCC97-L tumors (right; *t* = 3.293, *p* = 0.005). **b** The uptake of [^18^F]FLT in MHCC97-H tumors was lower than that in MHCC97-L tumors, but the difference was not significant (*t* = 0.870, *p* = 0.409). **c** The uptake of [^18^F]ACE in MHCC97-H tumors was higher than that in MHCC97-L tumors, but the difference was not significant (*t* = 0.923, *p* = 0.369).

MicroPET images of the subcutaneous tumor-bearing model are shown in Fig. 5. [^18^F]FLT had a higher radioactivity concentration in MHCC97-H and MHCC97-L mice compared with [^18^F]FDG and [^18^F]ACE. The T/NT ratio of [^18^F]FDG in the MHCC97-H xenograft (5.061 ± 0.874) was significantly higher than that in the MHCC97-L xenograft (3.923 ± 0.521; *t* = 3.293, *p* = 0.005). Although the T/NT ratio of [^18^F]FLT in MHCC97-H tumors (10.946 ± 2.472) was also higher than that in MHCC97-L tumors (10.149 ± 0.716), there was no significant difference (*t* = 0.870,* p* = 0.409). There was no significant difference in the T/NT ratio of [^18^F]ACE in MHCC97-H tumors (2.409 ± 0.229) and MHCC97-L tumors (2.331 ± 0.120; *t* = 0.923, *p* = 0.369).

MicroPET images of the metastatic model showed that the T/NT ratio of [^18^F]FDG in MHCC97-H tumors (22.671 ± 4.846) was significantly higher than that in MHCC97-L tumors (6.648 ± 2.098; *t* = 6.909, *p* < 0.001), whereas the T/NT ratio of [^18^F]FLT in the MHCC97-H (10.587 ± 3.876) and MHCC97-L (7.348 ± 1.862) tumors was not significantly different (*t* = 1.910, *p* = 0.088). In contrast, the [^18^F]ACE uptake of tumor was similar to that of surrounding liver tissue in metastatic models and there was substantial radioactive accumulation in the intestine, therefore it was difficult to draw a ROI of the tumor from the images.

### Quantitative RT-PCR Analysis of MMP9 and VEGFR-2 in MHCC97-H and MHCC97-L Tumors

mRNA expression of MMP9 and VEGFR-2 was examined by RT-PCR. Semi-quantitative analysis showed higher expression in MHCC97-H tumors than in MHCC97-L tumors for both MMP9 (2.761 ± 0.868 vs. 0.990 ± 0.439, *t* = 3.154, *p* = 0.034) and VEGFR-2 (3.257 ± 0.203 vs. 1.218 ± 0.584, *t* = 5.715, *p* = 0.005).

### Correlation Analysis

**Fig. 6:**
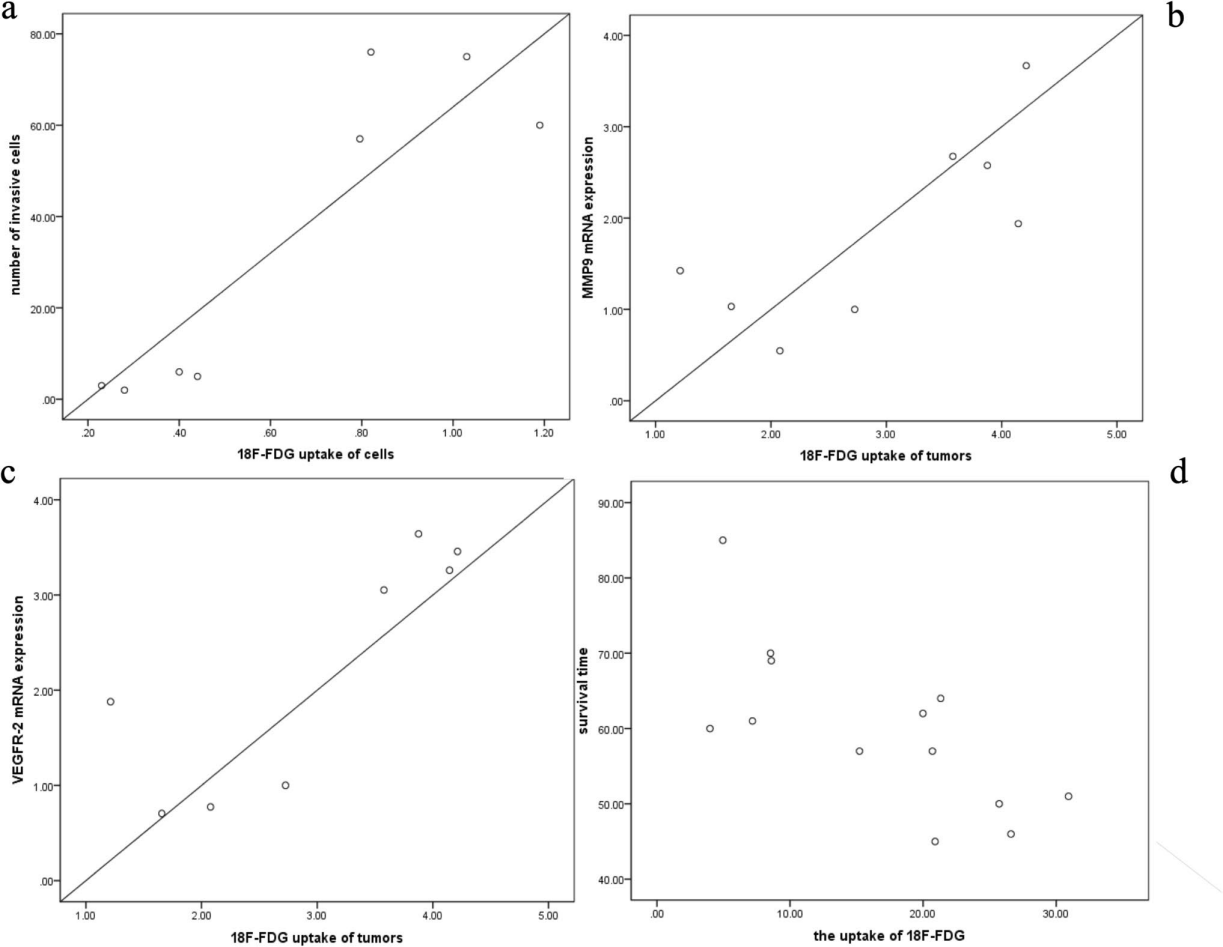
Correlation analysis of [^18^F]FDG uptake with cell invasion, tumor biomarkers, and survival in HCC models. **a** Correlation between invasion ability and uptake of [^18^F]FDG PET in HCC cells (*r* = −0.857, *p* = 0.007). **b** Correlation between MMP9 mRNA expression and the T/NT ratio of [^18^F]FDG PET in HCC (*r* = 0.770,* p* = 0.025). **c** Correlation between VEGFR-2 mRNA expression and the T/NT ratio of [^18^F]FDG PET in HCC (*r* = 0.830, *p* = 0.011). **d** Correlation between survival time and the T/NT ratio of [^18^F]FDG PET in HCC (*r* = −0.726, *p* = 0.005)

The in vitro uptake of tracer by cells correlated with their invasion and migration ability for [^18^F]FDG (*r* = − 0.857, *p* = 0.007, *r* = − 0.881, *p* = 0.004 respectively) and [^18^F]FLT (*r* = − 0.833, *p* = 0.010, *r* = − 0.786, *p* = 0.021) (Fig. 6a). In contrast, there was no correlation between [^18^F]ACE uptake and invasion or migration ability (*r* = 0.357, *p* = 0.385; *r* = − 0.333, *p* = 0.420).

In the in vivo assay, [^18^F]FDG uptake of tumors correlated with mRNA expression of MMP9 (*r* = 0.770,* p* = 0.025) and VEGFR-2 (*r* = 0.830, *p* = 0.011) (Fig. 6b, 6c). The [^18^F]FLT uptake of tumors also correlated with MMP9 (*t* = 0.911, *p* = 0.002) and VEGFR-2 (*r* = 0.883, *p* = 0.004) mRNA expression. In addition, there was a correlation between [^18^F]FDG and [^18^F]FLT uptake (*r* = 0.793, *p* = 0.019). In the metastatic model, [^18^F]FDG uptake in mice showed a significant negative correlation with survival time (*r* = −0.726, *p* = 0.005; Fig. 6d), whereas there was no correlation between [^18^F]FLT uptake and survival time (*r* = −0.217, *p* = 0.521).

## Discussion

Metastasis is one of the major therapeutic challenges in the disease course of HCC. Li et al. [16] have developed a stepwise metastatic human HCC model system that provides a platform for studies of the mechanism of HCC metastasis and searches for proteins, genes, and chromosomes related to HCC metastasis [17]. Similarly, most other studies on HCC metastasis and relative biological characters have been conducted at the cellular level. In contrast, there are limited data on whether differences in metastatic potential could be determined or predicted by in vivo imaging. The present study aims to determine whether the three PET tracers, namely [^18^F]FDG, [^18^F]ACE, and [^18^F]FLT, possess the ability to differentiate the characteristics of four hepatocellular carcinoma (HCC) cell lines under both in *vitro* and in *vivo* models.

There are some previous reports of dual-tracer PET/CT imaging for hepatocellular cancer and metastasis, but the data merely showed that dual-tracer PET/CT increased the sensitivity of PET/CT in HCC and had an complementary advantage in the evaluation of HCC metastasis compared with single-tracer imaging, and the authors did not integrate the uptake data of different tracers in HCC cells and analyze these data by statistic methods. In our study, we found that uptake of three tracers in four HCC cell lines was significantly different, and subsequently established a multitracer classification model to differentiate four HCC cell lines with different biological behaviors. The classification error rate with three features ([^18^F]FDG + [^18^F]FLT + [^18^F]ACE) was lower than that with two features ([^18^F]FDG + [^18^F]FLT, [^18^F]FDG + [^18^F]ACE, [^18^F]FLT + [^18^F]ACE), showing that multitracer PET better differentiated HCC cell lines with different biological behavior compared with dual tracer. Currently, a small number of studies have also reported the application of three tracers in the diagnosis and therapy of tumors. some tumors exhibit low FDG avidity, necessitating alternative radiotracers for accurate detection. Given the complexity of tumor biology, Intrapatient intermetastatic heterogeneity (IIH) and the variability of individual patient responses, it is imperative for us to explore alternative or complementary imaging modalities.The combined use of the three tracers can reveal the distinct biological characteristics of tumors from multiple dimensions and perspectives, which is expected to provide more comprehensive support for the diagnosis of liver cancer and other cancers.These imaging phenotypes can even provide robust evidence for the clinical practice of precision medicine[23–25].

Studies at the tissue level are more biologically and clinically relevant to disease than cell lines, and for such studies in HCC an in vivo model with metastatic potential is required. MHCC97-H and MHCC97-L cells were chosen for xenograft models because they have a similar genetic background but different metastatic potential. Li et al. [17] previously showed that MHCC97-H cells have a significantly higher rate of metastasis and a stronger invasion capacity than MHCC97-L cells. The clinical outcome and molecular biomarkers used in the present study could serve as the gold standard and lay the groundwork for further comparative analysis. We showed that the biological behaviors of the MHCC97-H and MHCC97-L tumors were significantly different. The growth of the MHCC97-H subcutaneous xenograft was faster than that of MHCC97-L in the same mouse. Metastatic incidence was significantly higher and survival time was significantly shorter in the MHCC97-H model compared with the MHCC97-L model. These results are consistent with previously reported findings for these two cell lines, indicating that cells with the same genetic background might indeed behave differently.

Unlike pathology-based measurements, PET, as a bioimaging-based method, is non-invasive and has good repeatability [26]. As PET tracers, [^18^F]FDG measures glucose metabolism in the HCC tumor, [^18^F]FLT assesses cell proliferation, and [^18^F]ACE is captured in the TCA cycle and can be metabolized into the membrane metabolic pathway. These biological modalities thus show various behaviours of the same tumor. In vivo microPET imaging of the subcutaneous tumor-bearing model revealed that the T/NT ratios of [^18^F]FDG in MHCC97-H and MHCC97-L were significantly different, whereas there was no statistical difference in the T/NT ratios of [^18^F]FLT in MHCC97-H and MHCC97-L. These findings are in accordance with the results for the spontaneous metastasis model and indicate that glucose metabolism is more active in an HCC cells with high metastatic potential than in those with low potential. This is consistent with what has been reported in a recent study of colon cancer by Malviya [27]. Clinical studies have proven that [^18^F]FDG PET is an advanced diagnostic imaging technique for detecting recurrence and metastasis due to its high sensitivity and specificity. However, there are limited studies that differentiate the metastatic abilities of HCC by [^18^F] FDG imaging. The results of the present study indicate that the T/NT ratio of [^18^F] FDG is valuable for differentiating the metastatic potential of HCC in vivo. But macrophages and immune cells also exhibited the highest glucose metabolism in tumor models [28]. Therefore, it can be inferred that the level of [^18^F] FDG uptake in the tumor model of this study, on the one hand, be related to the biological characteristics of the tumor, and on the other hand, be affected by the tumor microenvironment. There are limited reports about [^18^F]FLT or [^18^F]ACE imaging in HCC [9, 10, 13, 26]. In our study, No significant difference was observed in the uptake of [^18^F]FLT in MHCC97-H and MHCC97-L cells between in vitro cells uptake assay and the subcutaneous tumor-bearing model. Ho et al. [29] reported that HCC lesions with high 11C-acetate had no uptake of [^18^F]ACE, whereas Takemoto et al. [15] found moderate uptake of [^18^F]ACE in HCC and cholangiocellular carcinoma. In our study, [^18^F]ACE was first used to differentiate HCC models with different metastatic potential. We found that different HCC cells had different uptake of [^18^F]ACE, but there was no relationship between biological behaviours and [^18^F]ACE uptake. Moreover, there was no significant difference in [^18^F]ACE uptake between subcutaneous xenografts with high or low metastasis potential. The discrepancy in [1⁸F] ACE uptake between in vitro and in vivo settings is likely correlated with the tumor microenvironment or the expression of ACSS in cells. Therefore, [^18^F] ACE cannot be deemed superior to [^18^F] FDG as an oncologic tracer for evaluating the metastatic potential of hepatocellular carcinoma (HCC).

The MMP proteins are a family of important enzymes that degrade the extracellular matrix and are positively correlated with invasion/metastasis [30]. The difference in the metastatic abilities of MHCC97-H and MHCC97-L cells is associated with the secretion level of MMPs [31]. MMPs are also a significant predictive factor of recurrence after resection in HCC patients [32]; in particular, MMP9 is an important metastasis-related biomarker in HCC. VEGFR-2 is the primary receptor mediating the angiogenic activity of VEGF and the VEGF/VEGFR axis is recognized as an important regulator of tumor angiogenesis in HCC [33]. In the present study, the mRNA expression levels of MMP9 and VEGFR-2 were significantly different between MHCC97-H and MHCC97-L tumors. MHCC97-H tumors with high metastatic potential had higher expression of MMP9 and VEGFR-2 mRNA, and the expression of MMP9 and VEGFR-2 mRNA correlated well with the T/NT ratio of [^18^F] FDG in vivo; therefore, the uptake of [^18^F]FDG probably reflects metastatic potential via it exhibited a tendency toward positive correlation with MMP9 and VEGFR-2. Moreover, survival time was significantly shorter for the MHCC97-H model with higher [^18^F]FDG uptake than for the MHCC97-L model with lower [^18^F]FDG uptake (*p* < 0.05) and there was a significant correlation between [^18^F]FDG uptake and survival time (*p* < 0.05). These results suggest that [^18^F]FDG uptake might be a prognostic factor in HCC, which is not surprising because previous study have shown that FDG PET/CT was an independent biomarker for the estimation of disease-free survival in HCC patients through radiomics analysis [34]. [^18^F]FDG PET/CT also can predict overall survival of HCC patients before liver transplantation by utilizing a deep learning model (3D ResNet-18)[35]. These findings indicate that [^18^F]FDG PET is likely to be important not only in identifying tumors but also in determining their biological behaviors and prognosis [36]. Interestingly, the present study showed that HCC models with different metastatic potentials had different [^18^F]FDG uptake. [^18^F] FDG uptake was related to survival time, and MHCC97-H tumors with high expression of MMP9 and VEGFR-2 show higher [1⁸F]FDG uptake compared to MHCC97-L tumors with low expression of these two molecules. Although the mechanism is not clear, these results suggest that [^18^F]FDG could reveal more information on the biological behaviors of tumors than other two tracers.

The present study was limited by the relatively small sample size used. Further studies with more animals and other HCC metastasis models are needed to assess the relationships between tracer uptake and the biological character of HCC and the underlying mechanisms, and to verify the feasibility of a multitracer classification model in animal models with different biological behavior.

## Conclusion

A multiparameter (multitracer) classification model had superior ability to discriminate four HCC cell lines with different biological behavior compared with dual tracers or single tracer through in vitro cell uptake experiment. In both subcutaneous tumor-bearing models and metastatic models, [1⁸F]FDG uptake can distinguish between tumor models with high and low metastatic potential, and [1⁸F]FDG uptake can predict the survival time in metastatic models.

Data were analyzed using Tamhane’s T2 test. Values in parentheses represent *p*-values. * indicates *p* < 0.01.

All experimental animals were male mice. For all scans, the tracer injection dose was fixed at 500 μCi in a volume of 0.1 ml.

**a** In the MHCC97 H metastatic group (initial n = 8), all mice underwent [^18^F]FDG imaging; however, 4 mice died before [^18^F]F FLT imaging could be completed.

**b** In the MHCC97 L metastatic group (initial n = 7), all mice underwent [^18^F]FLT imaging, but 2 mice were lost to mortality before [^18^F] FDG imaging.

## Data Availability

The data of the study is available from the corresponding author on reasonable request.
